# Automated Vehicle Traffic: A Review of Operational Challenges, Infrastructure Requirements and Research Directions

**DOI:** 10.3390/s26041232

**Published:** 2026-02-13

**Authors:** Eleni G. Mantouka, Katerina Vakrinou, Konstantinos N. Christidis, Babis Magoutas, Akrivi Kiousi, Eleni I. Vlahogianni

**Affiliations:** 1 Department of Transportation Planning and Engineering, School of Civil Engineering, National Technical University of Athens, Zografou Campus, 5 Iroon Polytechniou Str., 157 73 Athens, Greece; 2 Frontier Innovations, 5 Laskaratou Str., 111 41 Athens, Greece

**Keywords:** Connected, Cooperative and Automated Mobility (CCAM), Operational Design Domain (ODD), Safe System Design (SSD), automated vehicle traffic

## Abstract

The evolution of Connected, Cooperative, and Automated Mobility (CCAM) systems represents a shift in transportation, potentially achieving benefits in efficiency, sustainability, and safety. Large-scale deployments of CCAM systems are, however, still constrained by fragmented Operational Design Domain (ODD) and limited infrastructure readiness. This paper reviews the state of the art regarding operational, infrastructural, and technological enablers for predictive and extendable ODDs. First, a literature review of existing definitions and ongoing standardization work is presented, focusing on gaps in the formalization and validation of ODD boundaries. Second, the influence of physical infrastructure elements on vehicle performance and safety is analyzed. Third, technological and organizational enablers, which include digital twins, data-driven simulation models, and governance frameworks, are discussed in depth as essential in adaptive and resilient CCAM operations. The review concludes that predictive and extendable ODDs require a data-driven and interoperable mobility ecosystem linking vehicles, infrastructure, and governance. Future research should focus on developing measurable indicators for infrastructure readiness, advancing simulation tools for dynamic ODD monitoring, and integrating human-in-the-loop systems for safe mixed traffic. Aligning these advances with Safe System Design and AI governance frameworks will enable scalable and trustworthy automated mobility.

## 1. Introduction

The evolution of CCAM systems has the potential to transform the developments in the transportation industry, since they offer significant benefits to efficiency, sustainability, and road safety [1,2,3,4]. However, the transition to automated mobility is not without challenges. A critical and often underestimated aspect of ensuring the successful integration of Connected and Automated Vehicles (CAVs) is the readiness of physical infrastructure to support their operational needs. Traditional road infrastructure, designed primarily based on human driver behavior, should now adapt to accommodate the unique requirements of vehicles operating under varying levels of automation [5].

The concept of ODDs plays a significant role in this integration. The Society of Automotive Engineers (SAE) defines ODD as the operating conditions under which a given automation system or feature is specifically designed to function, including, but not limited to, environmental, geographical, and time-of-day restrictions, and/or the requisite presence or absence of certain traffic or roadway characteristics [6]. The definition of the ODD can include physical infrastructure, operational constraints, objects, connectivity, environmental conditions, and zones [7], or scenery elements, environmental conditions, and dynamic elements [8,9]. Elements of the physical infrastructure or scenery elements, such as road geometry, pavement quality, signage, lighting, and maintenance, are integral to determining the boundaries of these ODDs.

When an Automated Driving System (ADS) departs its ODD, it requires input from a human to operate; thus, there exists a transition from ADS to human control that may compromise safety, as there is a period during which the vehicle may be under the control of neither, or it may lead to unexpected and unsafe behaviors [10]. Moreover, if the ODD constitutes a small subset of the overall driving space, then there will exist large parts of the latter where the ADS of an AV will not be functionally different from a set of manually operated vehicles, which will negate their safety and traffic-efficiency benefits [11]. Therefore, extending ODDs is essential for enabling the safe and efficient deployment of CAVs, particularly where the environment involves complex and dynamic conditions. Current ODDs are often restricted to controlled or highly simplified conditions, such as highways or geo-fenced urban zones, limiting actual scalability as well as public utility of automated mobility systems. To address these constraints, broader and more robust ODDs are needed, ones that allow AVs to handle real-world conditions such as bad weather, roadworks, and mixed traffic scenarios without frequent disengagements [12]. This is essential for augmenting both operational scalability and public acceptance of automated mobility. Expanding ODDs also supports continuous mobility and traffic safety by allowing vehicles to respond better to dynamic situations.

The operational context is the cornerstone to ensure the performance and safety of CCAM. The ODD, by design, essentially defines the operating environment for which a system is designed. On the other hand, authorities and road operators are responsible for operating the Physical and Digital Infrastructure (PDI), which is typically serving a large variety of vehicles and systems, with limited knowledge on the diverse ODD specifications, roadside equipment, potential Infrastructure-to-Vehicle (I2V) and Infrastructure-to-Infrastructure (I2I) communication requirements, and traffic management needs [13]. To answer the question of how safe an AV is, one should not only address the ODD used for shaping the functional boundaries of the CCAM system (top-down approach), but also the infrastructure, system-specific matters, safe and secure communication, traffic conditions, as well as environmental conditions, such as weather, and unexpected hazards (bottom-up approach) [14]. As stated by the CCAM partnership’s position papers, to fully exploit the CCAM potential, more complex traffic situations must be tackled, addressing mixed traffic situations with enlarged ODDs. This will require innovations in vehicle technologies, e.g., increased perception and control, as well as on augmented infrastructure with cyber-secure connectivity [15].

From a traffic engineering standpoint, the emergence of connectivity and automation in vehicles requires, apart from human-centered design, a provision for a systemic approach that integrates technology, infrastructure, and diverse road users. When it comes to the actual physical infrastructure and those that operate it, there is significant gap between the design principles and the actual usage of such infrastructure, coming from electromobility, automated traffic, etc.

**Figure 1:**
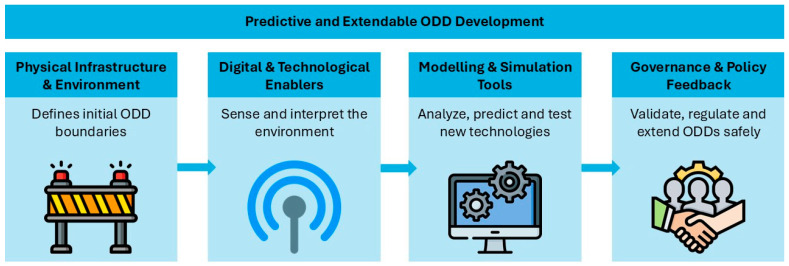
Workflow for predictive and extendable ODD development.

This review aims to bridge these gaps by presenting an integrated perspective on predictive and extensible ODDs. The motivation of this study is to understand how ODDs can be effectively defined, tested, and expanded through the coordination of technological innovation, infrastructure design, and institutional governance. Our objective is to synthesize the current landscape of standards, research, and deployment strategies to identify enablers of scalable and safe CCAM. While several reviews exist on automated vehicle technologies or safety assurance [4,16,17,18], few integrate the operational, infrastructural, and governance perspectives under a unified framework for predictive and extendable ODDs. To address this, we introduce a workflow for predictive and extensible ODD development (Figure 1), which guides the structure of this paper and breaks down predictive and extendable ODD development into four interconnected layers: physical infrastructure, digital and technological enablers, modeling and simulation tools, and governance and policy feedback. Each of these layers contributes iteratively to identifying, testing, and safely expanding the functional boundaries of AVs.

The remainder of the paper is structured as follows: Section 2 presents an overview of ODD standardization efforts, definitions, and related challenges. Next, Section 3 examines the state of the art in physical infrastructure requirements and adaptations for AV operation. Section 4 introduces the main technological and organizational enablers that can support predictive and extendable AV operations, including perception technologies, modeling tools, and AI governance. Lastly, in Section 5, the conclusions of the analysis are presented, summarizing findings and proposing directions for future research.

## 2. Operational Design Domain Standards and Challenges

As the foundational layer of the workflow for predictive and extendable ODD development, standards and definitions around the ODD establish the physical, digital, and environmental parameters within which automated systems can safely operate. This section explores how various standardization bodies define, structure, and validate ODDs, providing the base upon which digital enablers, simulation models, and governance practices are built.

### 2.1. Definition and Standardization

Given the rapid development of ADSs and the broader push towards CCAM, efforts to standardize the definition of ODD have intensified in recent years. Among the first in that direction is the SAE J3016 standard, which defines a categorization of driving into 6 levels of automation [6]. These levels are closely related to the definition of ODD, as each level above Level 2 implies a more complex and dynamic environment of operating conditions that the vehicle must be capable of handling without issuing a takeover request. Above Automation Level 3 the availability of a fallback-ready user should be considered as limited as it is not explicitly required. Vehicles that aim to prove Automation Level 4 need to be able to execute minimum-risk maneuvers without human input when they are outside their ODD, which generates in principle an additional ODD definition for minimum risk maneuvers that needs to cover most if not all of the Operational Domain (OD). A more formal initial effort to standardize the definition of ODD was conducted by [7], in a work supported by the U.S. National Highway Traffic Safety Administration (NHTSA). In that work six main categories of ODD attributes were identified: physical infrastructure, operational constraints, objects, connectivity, environmental conditions, and zones. These high-level categories have nested subcategories, creating a hierarchical model that captures both static and dynamic aspects of the vehicle’s environment, enabling a more in-depth understanding of the conditions under which an ADS may be used safely.

Similarly, ISO 34503:2023 [9] proposed a hierarchical framework for ODD definition with three levels. At the top-level classification, ODDs are divided into three domains, which include scenery elements, such as roads, lanes, and traffic furniture; environmental conditions, which include factors such as weather, lighting, and connectivity status; and dynamic elements, such as moving agents like vehicles, cyclists, and pedestrians. Mid-level classification proceeds to subdivide these domains to contextual factors such as zones and the drivable areas, which constitute scenery elements and offer a more comprehensive classification. At the low-level classification, quantitative specifications and thresholds in terms of detail are defined, e.g., zone type or driving lane width. Additionally, minimum requirements for ODD definition are identified, especially with regard to junction geometry and types of traffic control, as well as different ways to define environmental aspects. Proposed limits are given along with a provision for stakeholders to change them according to their implementation’s capabilities and requirements.

A generic ODD definition taxonomy is presented by [19], in which they collect and analyze a wide range of existing ODD classification approaches by standardization bodies and scientific literature. The proposed taxonomy consists of six primary categories: physical infrastructure (i.e., roadway geometry, signs, and surface), scenery (i.e., specific zones and geofencing), environmental conditions (i.e., rain, lighting), traffic conditions (i.e., density and users on the road), digital infrastructure (i.e., communication technologies), and vehicle capabilities (i.e., maneuverability and speed range). This taxonomy was tested and applied in a real-life scenario and found to be applicable in the development, evaluation, and safety assurance of automated systems such as the Bus Station Automated Service (BuSAS) system.

In parallel, ref. [20] introduced a formal mathematical model for the specification and definition of both the OD and the ODD of AVs. This attempt addresses the inconsistencies in earlier definitions by clearly differentiating the OD, i.e., the full range of physical, environmental, and traffic conditions in which vehicles operate, and the ODD, i.e., the limited subset optimized for the capabilities of a specific ADS. To describe the ODD, it is suggested to use simple and clear rules based on measurable characteristics (such as road type, speed, or weather). Also a method was proposed d to check in real time whether the vehicle is still operating within its ODD, based on what its sensors detect. If the vehicle goes outside of its allowed conditions, it can take safe fallback actions, such as slowing down or asking the driver to take over. This approach helps improve safety and makes it easier for engineers to design, test, and explain how and where automated vehicles can be used.

All in all, ODDs provide the foundation for the safe, efficient, and widespread deployment of CCAM systems. ODDs specifically define the environmental, infrastructure, and traffic conditions under which an Automated Driving System (ADS) can operate safely and reliably. Thus, ODDs set clear boundaries regarding where and when the vehicle can autonomously drive.

### 2.2. Scenario-Based Testing, Coverage, and Safety Metrics

As ADSs emerge, their safety under various circumstances has become a major issue. Scenario-based testing is one of the main methods applied to evaluate the safety of ADSs, utilizing realistic or challenging driving conditions, referred to as “scenarios”, to verify the performance of the system. One of the earliest approaches to testing scenarios was presented by [7]. Their framework identified seven generic ADS features to be tested, which roughly correspond to SAE automation levels 3 and 4, and specified three main testing environments: i. Modeling and simulation, ii. Closed-track testing, and iii. Open road testing, which can be carried out in free-flow highway traffic or mixed traffic conditions. Further standardization procedures include the ISO 34502:2022 framework for testing ADS safety on limited-access highways using scenario-based methods and the ISO 34504:2024 framework for the categorization of these scenarios into hierarchical trees using tags that correspond to the ODD categories from ISO 34503:2023 (such as road types or weather conditions) [9,21,22].

It is notable that existing scenario-based testing and safety evaluation approaches do not adequately address real-world testing in dense urban traffic, which is central to CCAM operations, particularly for applications such as fleets of connected and automated transit vehicles. Current frameworks rely predominantly on modeling and simulation, which, although essential for early-stage development and risk screening, are generally not regarded by regulatory bodies and road authorities, especially in Europe, as sufficient for direct real-world deployment. At the same time, real-life testing in mixed and dense urban traffic raises substantial safety concerns, limiting its feasibility at scale. This creates a significant gap between simulation-based validation and operational deployment. Bridging this gap requires a combination of more realistic, comprehensive, and well-documented simulation environments that better capture urban traffic complexity and human behavior, alongside more flexible regulatory approaches that safeguard public safety while enabling progressive CCAM development and ODD extension.

Furthermore, several scientific studies have attempted to find effective ways of designing and analyzing test scenarios for ADSs. One of the major challenges is defining how much real-world data is needed to ensure the system has been tested in all relevant conditions. To that end, ref. [23] developed a statistical method based on Gaussian kernel density estimation to analyze driver behavior from naturalistic driving data. This approach helps determine when enough data is collected to define the typical driving conditions and to identify risky behaviors. The second broad question is how to know when the testing can be stopped, i.e., when enough scenarios have been tested. Therefore, ref. [24] have addressed that using the Coupon Collector’s Problem (CCP), a classical probability model. By modeling scenario types as “coupons” and using statistical analysis and simulation, the number of samples that should be drawn so that one can be sure all possible types of traffic scenarios have been tested at least once is determined. This provides a data-driven way to justify ending testing and helps reduce unnecessary simulation runs or data collection.

A work by [25] examined to what degree pseudo-randomly generated simulation scenarios capture real-world traffic cases. A process was proposed to create scenarios through a step-by-step flowchart: first defining a route, then dividing it into smaller parts, designing possible scenes for each part, and finally producing complete scenarios from these elements. This method enables analyzing whether simulations are sampling enough of the scenario space relevant to autonomous vehicle safety. Further expanding on scenario development, ref. [26] introduced a methodology to quantify the cost-optimal usage of scenario generation approaches It was found that a very large number of scenarios needed to be recorded and/or generated in order to achieve high coverage, and that the cost escalated greatly as target coverage increased and allowed error rate decreased. This finding highlights the importance of balancing test coverage, cost, and resource constraints when planning scenario acquisition strategies. That work was extended by [27], by creating an approach to assess whether a scenario set is complete enough to support safety validation. Using goal-structured notation (GSN), they proposed a logical framework to justify the completeness of a scenario library, supporting both developers and safety assessors in making transparent safety claims.

Whether or not testing scenarios capture the ODD of an ADS entirely has also been addressed by [28], where a quantitative approach was introduced to evaluate ODD coverage by combining two components: scene prediction of traffic participants, and probabilistic risk assessment. Scenarios were evaluated according to their reliability, validity, sensitivity, and specificity. Simultaneously, ref. [16] proposed four coverage metrics for scenario database: (i) Tag-based coverage, which explores whether all the required scenario attributes (e.g., rain, road type, lighting) are represented, (ii) Time-based coverage, which ensures variation across different timeframes or durations, (iii) Actor-based coverage, which ensures a variety of road user types are included (e.g., cars, pedestrians, cyclists) and (iv) Actor-over-time-based coverage, which observes how actors behave and interact over time within a scenario. Their findings indicate that most existing scenario datasets have partial ODD coverage unless specifically designed to do so. They emphasize how the proposed metrics can be employed to identify such gaps and guide targeted data collection to improve scenario representativeness.

To determine how safe ADSs are under different conditions, many safety metrics have been developed. These metrics help to make decisions regarding whether the vehicle behaves safely in both common and critical scenarios. Among the most popular safety metrics is Time-to-Collision (TTC), first introduced by [29], which estimates the remaining time until a collision would have occurred if both vehicles continue at their current speeds. A work by [30], introduced the Instantaneous Safety Metric (ISM), which uses vehicle acceleration maps and physical characteristics to calculate possible vehicle trajectories. It simulates different interactions with other vehicles and then classifies the severity of potential collisions. Another approach by [31] developed the Responsibility Sensitive Safety (RSS) method, which formalizes five “common sense” rules, such as keeping a safe distance and reacting properly to other vehicles. These rules are used to determine whether the behavior of the system is safe across different driving situations. Moreover, ref. [32] proposed the Model Predictive Instantaneous Safety Metric (MPrISM), which measures how close an ADS is to a collision by solving an optimization problem. This metric works in real time and considers both lateral and longitudinal movements, making it more comprehensive than simpler metrics such as TTC. An extension to safe stopping distance used in road design standards is the Minimum Safe Distance Violations (MSDV), which determines if the vehicle is maintaining a minimum safe distance from others [33]. Recently, ref. [34] proposed a wide variety of metrics termed as criticality metrics that quantify how dangerous a given traffic scenario is, based on time gaps, distance, and speed differences. Such metrics are especially valuable for calculating how close an ADS came to a critical situation, even if the collision did not take place [35,36]. Last, ref. [37] defined the repulsion force inversely proportional to a TTC-related metric to account for mixed vehicle and pedestrian interactions at shared space roads.

A more recent contribution to safety evaluation comes from [38], where a safety metric is presented that aims to provide a guaranteed and unbiased safety assessment, even with a limited number of scenario samples. The authors highlight that many modern safety metrics either rely on incomplete sampling or are focused on rare crash outcomes. To address this, they introduce a new method that combines concepts such as the α-shape, which determines the boundaries of the observed vehicle actions, and the ε-almost invariant set, which signifies operating situations in which the system is safe with high probability [39,40]. This shifts the focus from counting events to analyzing the shape and boundaries of safety-relevant behavior, making it suitable for comparing different ADS systems under varied ODDs. Additionally, their framework includes both leading safety measures, i.e., those that measure system behavior before harm occurs, and lagging indicators, such as crashes or near crashes [10,41,42].

Despite their importance, current simulation-based testing frameworks exhibit limitations in modeling extremely rare events and complex human behavioral uncertainty. Rare but safety-critical scenarios, such as highly atypical interactions or compound failures, are difficult to capture due to their low probability of occurrence and the reliance of simulations on predefined scenario spaces or historical data [43,44]. Moreover, human behavior in mixed traffic environments is inherently stochastic and context-dependent, which limits the ability of simulation models to fully represent variability in decision-making, risk-taking, and non-compliant behavior. As a result, while simulation remains a critical tool for ODD extension and early-stage safety assessment, it cannot alone provide sufficient assurance for real-world deployment, highlighting the need for complementary validation approaches and cautious interpretation of simulation-based results.

Additionally, human factors remain a critical element in ODD definition and safety validation, especially in mixed-traffic scenarios where human drivers and ADS-equipped vehicles interact. Key aspects include driver readiness to resume control, takeover performance, and the design of Human–machine Interfaces (HMIs) that support intuitive and timely driver responses [45]. Studies show that inadequate HMI design or unclear transition protocols can lead to delayed or unsafe takeovers, underscoring the need to integrate behavioral data and user interaction models into ODD testing frameworks and scenario generation [46].

Table 1 summarizes recent research on scenario-based testing and safety-related metrics for the evaluation of CAV traffic.

### 2.3. Representative Mixed-Traffic Pilot Deployments and ODD Management

While simulation and testing provide important insights, real-world pilot deployments show how ODDs are managed in practice. Several mixed-traffic pilot deployments reported in the scientific literature provide useful insights into how ODDs are defined, constrained, and managed in real-world conditions. These pilots typically rely on conservative ODD definitions, gradual extension strategies, and close interaction with regulatory authorities in order to ensure safe operation in environments shared with human drivers and vulnerable road users.

Urban automated vehicle pilot deployments operating in mixed traffic have been documented in multiple studies, particularly for low-speed shuttles and urban automated driving trials. Such pilots commonly define their ODD through strict spatial constraints (e.g., geofenced urban areas), limitations on road types and intersection complexity, speed restrictions, and exclusion of adverse weather conditions (e.g., rain or low visibility) [47,48]. ODD management strategies reported in these studies emphasize continuous system monitoring, predefined fallback behaviors such as minimum-risk maneuvers, and incremental ODD expansion based on accumulated operational experience and safety validation outcomes rather than purely simulation-based evidence.

Highway-based truck platooning pilots provide a complementary mixed-traffic example, where automated vehicles interact with human-driven traffic under more structured conditions. The literature shows that these pilots typically restrict their ODD to limited-access highways, predefined speed ranges, controlled headways, and specific infrastructure characteristics, such as bridge capacity and ramp geometry [49]. ODD management in these cases focuses on maintaining stable operating conditions through platoon size limitations, spacing control, and integration with traffic management measures, rather than rapid ODD expansion [50]. Reported studies highlight that such constraints are essential for managing safety risks related to infrastructure loading, cut-in maneuvers by human drivers, and communication reliability.

### 2.4. Fragmentation and Interoperability Challenges in ODD Standards

Given the different and continuously evolving standards from multiple jurisdictions (USA, EU, global under ISO, etc.), as well as the multitude of specialized standards and guidelines addressing specific topics of CCAM, such as those developed by ETSI, it is clear that there does not exist a globally agreed-upon framework to enable the transferability of specific applications from one jurisdiction to the other. In practice, the absence of a globally harmonized framework limits the transferability of CCAM applications between jurisdictions and often requires additional effort to demonstrate compliance with multiple, partially overlapping standards. While this review places stronger emphasis on European standardization efforts, parallel approaches in other regions, such as SAE- and NHTSA-led frameworks in North America and pilot-driven regulatory models in East Asia, also play an important role in shaping ODD definitions and CCAM deployment strategies.

These challenges are further exacerbated by a mismatch between technology research and development, commercial deployment, and standards development and adoption timelines. While research activities and pilot deployments advance rapidly, driven by both public and private investments, standards development and adoption typically follow longer cycles, making them quickly out of date and obsolete.

For example, while ETSI standards such as [46] provide detailed guidelines for collaborative perception and maneuvering, they are not reflected in ISO 34503:2023 [9], despite the fact that they are critical to the development of CCAM. Facilitating cross-standard and -jurisdiction compatibility and the wider, faster adoption of common standards and guidelines will enable faster application development and greater transferability at lower cost. Additionally, a proactive approach by standardization bodies, specifically ISO, by engaging researchers and developers in the standards development process can accelerate the turnaround of standards to keep them closer to date and encourage standards adoption.

In addition to the above, standards such as ISO 34503:2023 [9] could benefit from expansion and elaboration on specific CCAM applications, such as Vehicle-to-Infrastructure (V2I) and Vehicle-to-Everything (V2X) communication conditions and standards, the status and availability of collaborative perception and maneuvering services, and collaboration with actors such as traffic managers, transit service providers, and emergency services.

## 3. State of the Art on Physical Infrastructure for AVs

**Figure 2:**
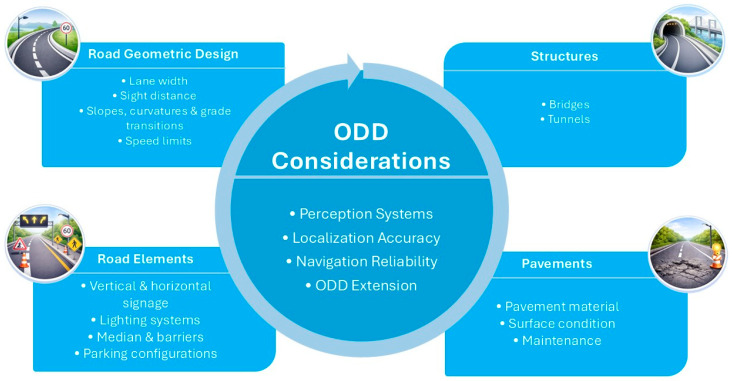
Physical infrastructure components influencing ODD considerations for automated vehicles.

The definition and standardization of ODDs provide the conceptual boundaries within which automated vehicles can operate safely. However, these boundaries are constrained and potentially expanded by the characteristics of the physical infrastructure. As outlined in the first component of the predictive and extendable ODD development workflow, physical infrastructure and environment serve as the foundational layer that defines initial ODD boundaries. The integration of AVs into transportation systems requires an understanding of the physical infrastructure elements that influence their functionality and safety. From the review of the existing literature, several key categories of physical infrastructure have been identified as crucial to supporting AV operation. Figure 2 illustrates the main categories of physical infrastructure that affect ODD definitions and AV performance, showing how they connect to broader system aspects like perception, localization, and navigation reliability.

### 3.1. Geometric Road Design

Guidelines in road geometric design principles and manuals have evolved through practical experience and are based on human driver characteristics and limitations. The integration of AVs might change the way roads are shaped, as some of these limitations might lose their importance and be replaced by the automated features. Advanced perception systems that integrate Global Navigation Satellite Systems (GNSS) with dead-reckoning algorithms are essential to maintain precise navigation within these environments. One of the most discussed parameters is the stopping sight distance that traditionally depends on human perception-reaction time. Studies have confirmed that AVs can significantly reduce perception-reaction time from 2.5 to 0.5 s, which corresponds to reduced stopping sight distance and decision sight distance needs [5,51,52]. These changes allow for shorter crest and sag curves, which can reduce earthworks and construction costs [5,53].

In addition, sensor height replaces driver eye height in calculations, making it possible to have shorter vertical curves [51]. For crest curves, LiDAR sensors positioned around 1.7–1.84 m enable shorter k-values, which refer to the rate of vertical curvature, and curve lengths, while for sag curves, the wider field of view of LiDAR reduces reliance on headlight angles [5,51]. Similarly, horizontal curves can be redesigned to improve sensor visibility and AV localization, even when the minimum radius cannot be altered due to dynamics and discomfort limitations [52,54].

From a lane width perspective, high-precision lateral control of AVs enables the possibility of reducing lane widths, with many studies suggesting minimum lane widths of 2.4 m to 2.75 m based on the automation level and the environment of operation [55,56,57]. While narrower lanes may increase road capacity, they may present a challenge to Level 2 AVs that rely on visible edge markings [52,54]. For full automation, studies propose using dedicated narrower lanes or minimum widths of 2.7–2.75 m for stability and minimizing transition errors [56].

Slope and grade transitions are also addressed in the literature. Slopes with steep inclines (>8%) would negatively impact Av operations and would require continuous and optimized vertical alignment to avoid sensor malfunction or power failure in shuttles and small AVs [55]. Smooth continuous-curvature paths are recommended to enable trajectory planning and tracking accuracy, especially at higher speeds [56].

Lastly, speed limits for AVs are expected to evolve in response to the unique operational capabilities of AVs. Literature suggests that traditional traffic calming features such as speed bumps and static limits will not be needed in highly automated environments and can be replaced with more dynamic, AV-compatible designs [54]. As AVs leverage sensor and software functionalities to adjust their behavior in real time, several studies propose variable speed limits depending on different use cases, e.g., low-speed shuttles versus highway vehicles, while also considering performance under adverse conditions. For instance, refs. [52,53] state that current AV systems perform reliably under conditions with a maximum speed limit of 80 km/h, and the optimal performance is generally obtained in the range of 70–90 km/h, considering sensor precision and lane-keeping ability. International standards reinforce these observations. ISO recognizes speed range as an essential ODD variable, utilizing ranges like low speed (≤10 km/h), medium speed (10–50 km/h), and high speed (>50 km/h) to differentiate between system functionalities [9].

### 3.2. Special Road Geometries and Infrastructures

The literature focuses on two main special road geometry components, tunnels and bridges. Tunnel configurations face challenges with the integration of AVs, mainly due to their often weak or lacking GPS signal. To address the weak GPS signals, wireless radio beacons or physical landmarks with sensor reflectors can be used to support and increase the positioning accuracy of AVs [53]. Also, advanced positioning systems with GNSS and Dead Reckoning are needed to maintain navigation precision when GPS signals are unavailable [56]. Another area that could affect the operation of AVs in tunnels is the low-light conditions. Consistent tunnel lighting, particularly at entrances and exits, reduces abrupt luminance transitions that negatively affect AV perception systems [54]. Additionally, the use of high-contrast lane markings designed for low-light conditions ensures that AVs’ vision-based systems can reliably detect road boundaries and maintain lane discipline. Finally, road surface modifications, such as the integration of Bluetooth/NFC-based guiding systems in pavement surfaces can potentially improve AV navigation in areas where GPS signals are poor, such as tunnels and urban canyons [5].

Bridges are an essential part of road infrastructure for AVs, particularly in the context of AV truck platooning. Existing bridge structures may be inadequate to handle the concentrated load effects of closely spaced truck platoons. Structural recalculations are necessary, especially for long-span bridges, to assess the impact of reduced headways and increased live-load demands [58]. Studies have shown that closely spaced platoons lead to elevated bending moments and shear forces, requiring updated design standards and retrofitting strategies [58,59]. One of the primary mitigation approaches includes regulating truck spacing, with findings suggesting that increasing platoon spacing from 9 to 12 m can reduce bridge stress and enhance safety by up to 68% [5]. Additionally, implementing traffic management systems to limit the number of trucks crossing a bridge simultaneously has been proposed as an effective strategy.

For newly constructed bridges, design adaptations are recommended to consider the dynamic loads imposed by AV platooning [54]. That involves using high-strength materials, reducing span lengths, and incorporating load-distribution mechanisms to handle concentrated forces safely. Safety margins need to be enhanced to account for variations in truck configurations and platoon sizes. International standardization of bridge design is also necessary to reduce the differences in guidelines across different countries.

Moreover, existing bridge evaluation frameworks indicate that older bridges designed using AASHTO’s pre-2000 Load Factor Design (LFD) or Allowable Stress Design (ASD) methodologies may not withstand the increased live-load demands of truck platoons, while in contrast, more modern bridges designed under Load and Resistance Factor Design (LRFD) specifications generally perform better but still require targeted structural enhancements in specific conditions, such as long-span configurations [59]. A work by [60] has applied Monte Carlo Simulation (MCS)-based reliability analysis and fatigue assessments and highlighted the importance of structural monitoring, particularly for steel bridges that may experience accelerated fatigue damage. Additionally, researchers have developed prioritization metrics for bridge maintenance, combining load ratings and bridge condition factors to classify bridges from low to high priority for intervention [59].

### 3.3. Pavement Design, Materials, and Maintenance

AVs operate with highly precise lateral positioning, thus leading to narrow wheel wander, which results in channelized traffic and localized pavement stress. This contrasts with human-operated vehicles, where lane wanderings tend to help distribute the wear more evenly over the pavement surface [52]. Studies have shown that narrow wheel wander increases rutting depth by 175% compared to traditional traffic flow, having a significant impact on pavement life [61,62]. Furthermore, simultaneous multiple load applications from AVs and truck platoons with short following distances have the capacity to increase fatigue cracking and pavement degradation, particularly in urban roadways where lane width is narrower [54].

With that additional wear from AVs, high-stiffness, deformation-resistant materials are necessary. A work by [5] suggests incorporating strengthened materials for wheel paths that are especially designed to resist rutting and cracking because of the highly precise movement. Moreover, the employment of high-rutting-resistant asphalt and concrete mixture has also been suggested to better resist concentrated loading by AVs [52]. Other advanced materials, such as energy-harvesting pavements and magnet-embedded asphalt have also been explored to see if they can facilitate AV navigation and charging systems [61]. These materials would not only enhance AV performance but also extend pavement life. Other technologies are the development of rut-resistant asphalt that can distribute stress more effectively, using stiffer base and subbase layers to disperse concentrated loads. Furthermore, pavements can be thickened in high-traffic AV lanes to help absorb concentrated stresses and delay fatigue cracking [63]. Lastly, improving skid resistance and maintaining uniform lateral wheel loading across the lane can help reduce pavement damage and extend service life [54].

To also mitigate the effect of AV-induced pavement degradation, several AV design adjustments and speed considerations have been proposed. One of them is lateral control optimization, where AVs are set to implement pre-specified lateral wander behaviors, e.g., double-peak Gaussian distributions, to redistribute loads along the lane width and reduce rutting and fatigue [63]. AV technology has the potential to enable reduced vehicle spacing and higher-capacity roads, which would also influence pavement wear. Studies have shown that the potential to maintain higher traffic speeds can reduce rut depth by up to 50%, as lower speeds lead to longer pavement loading times and deeper rutting [56]. However, speed optimization should balance road safety and pavement management.

The localized loading patterns introduced by AVs and platoons increase the importance of pavement maintenance for sustaining reliable operation. AVs, in contrast to human drivers, are heavily dependent on road markings, signage, and pavement conditions for navigation and employ sensors such as cameras, LiDAR, and radar to interpret their surroundings [64]. Declining road conditions, such as faded lane markings, potholes, or rough road surfaces, can disrupt these sensors, resulting in potential operational issues. Recent research highlights the necessity of a proactive approach to road maintenance that includes smart technologies and predictive strategies to support AV-compatible infrastructure [65,66].

Studies emphasize the importance of routine upkeep of road markings, which serve as a primary guidance system for AVs. Regular repainting and the use of newer materials that have improved reflectivity and durability are recommended to enhance machine vision compatibility. High-speed roads, intersections, and areas with sharp curves are critical to maintain since visibility of lane markings in these road features is essential for safe AV navigation [55]. Furthermore, ref. [67] highlights that while AVs will primarily operate on existing road networks, they require a defined maintenance standard to ensure reliability. Besides road markings, pavement conditions also affect vehicle stability and sensor accuracy, particularly for LiDAR and camera-based perception systems. Transparent, wet, or light-absorbent surfaces should be avoided, as they can interfere with AV localization. Similarly, sandy or debris-laden roads pose challenges, as AV sensors may misinterpret dust particles as obstacles. To address these concerns, experts recommend improving road surfaces to meet International Roughness Index (IRI) standards, ensuring even pavement and minimizing defects that could interfere with automated driving [68].

Modern road maintenance is moving from reactive repairs to predictive and proactive actions, with the help of data-driven approaches and emerging technologies. Connected vehicles and AVs themselves serve as mobile sensors, continuously scanning road conditions and transmitting data on potholes, cracks, and surface wear [68]. This data could be integrated into predictive maintenance, allowing authorities to schedule timely interventions ahead of time before issues escalate. Additionally, high-resolution cameras and AI-based image recognition can be used in drones to detect early signs of road damage and arrange repairs accordingly. This approach minimizes unexpected failures, ensuring uninterrupted AV operation and reducing the need for disruptive emergency repairs.

### 3.4. Vertical and Horizontal Signage

Road markings are essential for AV navigation by establishing clear lane boundaries for machine vision. High contrast, retroreflective materials enhance visibility. A minimum retroreflectivity of at least 150 mcd/lux/m^2^ during dry weather and 35 mcd/lux/m^2^ when wet to enhance AV detectability is suggested [69]. Standardized lane markings, typically 150 mm wide, improve AV visibility, while contrast striping of 5 cm and a 3-to-1 contrast ratio further improve it, especially at night or during poor weather conditions. Continuity and consistency in road markings are required to minimize AV perception errors, particularly at intersections and ramps [57]. Ghost markings, which are worn-out or outdated lane markings, can interfere with AV lane detection, requiring efficient removal procedures [5]. Solid white lines should also be utilized where strict compliance with lanes is required, and dashed lines should be placed carefully to prevent AV misinterpretation [55]. Irregular or inconsistent markings, particularly in construction zones, can confuse AV lane-keeping systems and should be avoided [67].

Traffic signs provide a safe and efficient AV navigation through the provision of standardized and clear information for machine-vision systems. To prevent confusion, the standardization and harmonization of traffic sign design, placement, and meaning must be ensured on a global scale [55]. Proper sign geometry and placement at standardized heights further improve machine readability while avoiding obstructions from roadside infrastructure, vegetation, or other vehicles [5]. Retroreflective materials enhance visibility, particularly in low-light or adverse weather conditions, so that signs remain perceivable by AV sensors [69].

Digital and dynamic signage presents both opportunities and challenges. While connected AVs can rely on digital map systems for real-time sign updates, ensuring the accuracy and reliability of such systems is a priority. Similarly, dynamic LED-based signs, though useful in providing real-time updates, may be less readable for AV cameras compared to static signage due to variations in brightness and refresh rates [67]. Also, upgrading Variable Message Sign (VMS) systems is necessary, as many traditional VMS signs were designed for human readability rather than machine interpretation [54].

### 3.5. Road Elements

The use of barriers and gates in road design is critical to the safe introduction of AVs. The medians and barriers should be maintained on high-speed roads to prevent head-on collisions and provide recovery areas for vehicles that are out of control, particularly during the transition phase when both AVs and manually operated cars are present on roads [70]. However, as the penetration rate increases safety buffers and physical divisions between opposing traffic directions can be reduced or removed altogether, optimizing available road space for additional lanes, sidewalks, or bicycle paths [71]. Furthermore, traditional flexible median barriers, i.e., wire rope barriers, may not be suitable as AV sensors may have trouble detecting small objects, and thus barriers with more detectable features need to be utilized [72]. Concrete safety barriers on high-speed roads give a distinct lane separation and enhance AV detection and perception [56].

Medians should be wide enough to allow errant vehicles additional recovery time and space to reduce the impact of crashes. Additionally, median materials and textures should be identifiable by CAV vision systems so as not to be confused with adjacent road lines or shoulders [56].

Aside from static barriers, shoulders and safe stopping areas are needed for AV operations. It is necessary to have broad paved shoulders to allow AVs to stop in conditions of minimum risk, particularly where the roads are narrow, and where emergency stopping refuges at regular intervals must be provided [53]. Similarly, Emergency Refuge Areas (ERAs) have been proposed to provide safe stopping places for AVs in the event of failure or adverse environmental conditions. Research suggests ERAs should be at least 100 m long, with further research needed to determine the optimal ERA size and spacing distances to meet AV needs [5].

Lighting is crucial for the safe and efficient operation of AVs, particularly in urban and suburban environments where sensor detection is compromised by low light and adverse weather conditions. Good and uniform quality lighting is required to assist in ensuring that AV sensors, including cameras and LiDAR, can effectively detect road markings, traffic lights, and pedestrians. Streetlights need to be arranged in a manner that would form fewer shadows and ensure the even distribution of lighting on road surfaces and pedestrian crossings to eliminate the risk of misinterpretation by AV systems [56]. There should also be proper streetlights on roads that are expected to be used by cars with lane support systems, which would improve their functional reliability at dawn and dusk times [57].

The presence of AVs is both a challenge and an opportunity for parking infrastructure. With on-street parking potentially obstructing traffic flow and AV navigation, some studies recommend limiting or removing on-street parking in congested areas to enable a smoother traffic flow [54]. Autonomous valet parking systems and blocking strategies, where AVs park in a manner that temporarily blocks other vehicles but can automatically change positions, are being suggested as solutions to optimize parking space utilization. The arrival of autonomous valet parking offers a solution by allowing AVs to drop off passengers at their destination points and afterwards park away from there, reducing the demand for parking spaces and lowering traffic within urban centers [73]. In addition, research suggests that AV-adapted parking layouts could increase capacity by up to 2.5 times compared to conventional designs, demonstrating significant potential for reducing the urban parking footprint [5,74,75].

Table 2 summarizes the main findings of the literature review on physical infrastructure elements that influence AVs operations and ODDs.

## 4. Enablers of Predictive and Extendable AV Traffic Operations

**Figure 3:**
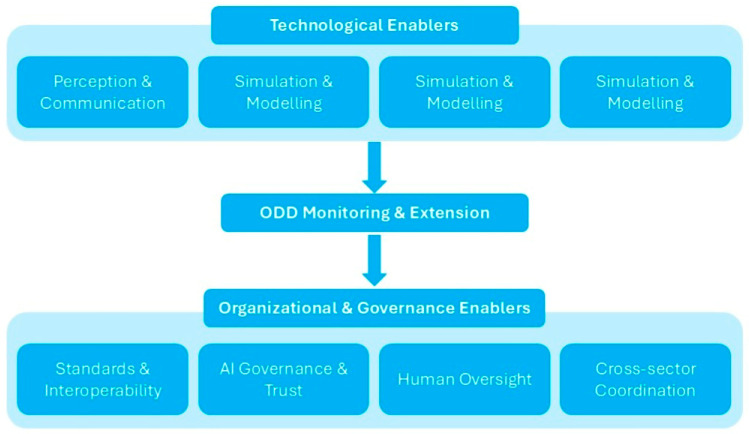
Overview of technological and organizational enablers that support ODD monitoring and extension.

While physical infrastructure defines the environment within which AVs can operate, it alone cannot ensure adaptability and resilience to such complex and dynamic driving environments. To address this gap, technological and organizational enablers that enhance perception, simulation techniques, coordination, and governance across all elements of the mobility ecosystem should be analyzed in alignment with the workflow for predictive and extensible ODD development, which structures these enablers as interconnected layers supporting scalable and safe CCAM operations. Figure 3 highlights the flow of influence from technological functions to system-level oversight and regulation, illustrating the dynamic interplay that enables predictive and adaptive ODD management in real-world deployments.

### 4.1. Technologies to Enhance Collective Perception

Allowing for dynamically evolving ODDs relates to high-resolution perception capabilities of the vehicle sourcing from monitoring in real time factors such as traffic conditions, weather, terrain, and road features and geometry, as well as rules of engagements, geographical and cultural variability, etc. These factors, linked to objects and events, are generally covered by the term Object and Event Detection and Response (OEDR) [77]. The detection of such elements is crucial for predictive ODDs, mainly through data-driven prediction models, to forecasting potential risks in driving or traffic situations that entail reduce safety levels.

To this end, real-time data provision is necessary. These data are sourced from the vehicle, the infrastructure and the users [78,79]. Novel technologies that need to be integrated to the existing ecosystem of data collection for traffic related purposes and increase resilience and performance of systems are the Global GNSS enhanced with 5G, inertial to support high-definition mapping and localization in challenging transport infrastructure environments (e.g., tunnels) and Digital Twins (DTs) in the highly detailed digital replicas of physical mobility systems, which can be vehicles, infrastructure, or entire urban transport ecosystems [80,81,82,83]. These technologies acting inside a decentralized data management framework can establish the missing links between vehicles, road users, and infrastructure at both an operational and organizational (governance) level and allow for real-time ODD extendibility through the continuous monitoring, updating (vehicle and infrastructure state), and management of the transport ecosystem’s vulnerabilities. Evidently, decentralized data management may address challenges with respect to privacy, security, and compliance, with the exponential increase in the data; technologies such as blockchain and federated learning can assist ensuring privacy and security of AV traffic [84].

Moreover, recent literature focuses on complementing the ODD through Remote Operations [85]. Although there exist many different remote operation functions and technological advancements, several challenges persist related to planning and design of the physical and digital infrastructure, human operator awareness and operational readiness, efficiency and trustworthiness, legal, business, and governance aspects, and societal awareness and acceptability. It should be noted that that research on the integration with Traffic Management Centers (TMCs) is limited. Interdisciplinary research is needed towards delivering Human-in-the-Loop technologies across system design and operation to improve the safe traffic operation of AVs and build trust, equity and long-term sustainability of remote operation [86,87].

Building on these data and sensing capabilities, collaborative perception and cooperative maneuvering enable vehicles and infrastructure to share situational awareness and coordinate actions in real time.

### 4.2. Collaborative Perception and Maneuvering

As CAVs begin to operate in more complex environments, the limitations of on-board perception systems become more apparent. Factors such as heavy traffic, bad weather, or objects blocking the view can make it harder for AVs to detect dangers or make reliable decisions. To address these limitations, researchers and policymakers have focused on collaborative perception to improve safety and expand ODDs. Collaborative or collective perception or awareness is the enhancement of situational awareness of an AV by sharing information with its environment. More specifically, it involves the transmission of an Intelligent Transportation System Station’s (ITS-S) perception to a vehicle via V2X communication. The transmitted information may include static information, such as road elements and signs, or dynamic information, such as other traffic agents. This information can be accessed from Roadside Units (RSUs), offering an external perception, or by leveraging the collected data from the AVs themselves.

Efforts to standardize collaborative perception and awareness have been made by the European Telecommunications Standards Institute (ETSI). One of the earliest documents, ETSI TR 102 638 V outlines the basic applications of V2X communication for enabling vehicles and infrastructure to share information in an attempt to make roads more efficient and safe [88]. Building upon this, ETSI EN 302 637-2 defines the Cooperative Awareness Service (CAS), which uses Cooperative Awareness Messages (CAMs) to enable vehicles and infrastructure to broadcast their location, speed, and status [89]. More recently, ETSI TS 103 900 updated the technical specification of CAMs to improve their use in different kinds of ITS-S, including RSUs and vehicles [90]. For more advanced capabilities, ETSI also defines the Collective Perception Service (CPS) in ETSI TS 103 324 [90]. CPS allows infrastructure and vehicles to communicate what they perceive, e.g., objects, obstacles, or pedestrians, beyond their line of sight. Interoperability among CPS implementations is facilitated by ETSI TS 103 926, which contains testing procedures to ensure different CPS-equipped devices can interoperate with one another successfully [91]. Finally, ETSI TS 103 900 and ETSI TS 103 926 together form a foundation for building cooperative and scalable CAV systems that can operate efficiently and safely in a shared road environment [90].

Collaborative perception is especially useful for spotting vulnerable users, e.g., pedestrians and cyclists [92,93,94]. Additionally, collaborative maneuvering is an essential part and advantage of CCAM systems as it can improve traffic flow and safety by enabling vehicles to communicate with each other in real-time, beyond the limitations of the traditional road rules followed by human drivers. This coordination is important for scenarios such as platooning, merging, or turning at intersections [95]. Cooperative maneuvering strategies can be categorized into implicit maneuvers, in which the vehicles broadcast their paths without expecting direct responses [96,97,98,99], explicit maneuvers, in which vehicles negotiate actions with each other through communication protocols [100,101,102] and emergent maneuvers, in which coordinated behaviors occur because of local interactions between vehicles [103]. The relevant literature is summarized in Table 3.

As shown in Table 3, much of the existing literature focuses on collaborative maneuvering at a theoretical or algorithmic level, while collaborative perception, despite being critical for traffic-dense, occlusion-rich urban environments relevant to CCAM, remains comparatively underexplored in real-world settings. This imbalance is largely driven by practical and technical constraints that are difficult or costly to overcome. Deploying RSUs for collaborative perception requires permits that are often time-consuming and expensive to obtain, particularly in the European context due to privacy legislation and public concerns. In parallel, setting up simulation environments that realistically capture both multi-vehicle automation and urban communication constraints is complex, as it requires the integration and validation of multiple driving, traffic, and telecommunications simulators. These challenges are compounded by the limited feasibility of real-world testing in dense urban traffic, where a substantial gap persists between simulation-based experimentation and regulatory approval for live testing. Together, these limitations help explain why collaborative perception remains insufficiently validated in practice, despite its recognized importance for extendable ODDs. The ability to simulate coordinated perception and maneuvering under complex conditions directly informs the traffic modeling and management strategies introduced in the next section.

### 4.3. Models and Simulation Tools for Traffic Management

From a modeling perspective, delivering predictive humanized “driving” models tends to be quite complex, given the variability of both infrastructure and mobility patterns [116]. Taking a physics-based approach by leveraging knowledge sourced from the vast body of literature on traffic flow theory and research on microscopic traffic modeling can reduce model complexity, improve the accuracy of predictive ODDs, and result in high acceptability through humanizing AV driving [117]. Microscopic traffic models can be enhanced with data-driven behavioral mechanisms, leveraging technologies such as quantum machine learning (ML) [118], reinforcement learning [119], GenAI [120], for realistic traffic representation in automated and shared-space environments, especially under safety-critical conditions [37,121]. Moreover, causal representation techniques for generalizing to new scenarios using on-the-fly test-time refinement are required [122]. This new hybrid form of modeling will combine knowledge-driven and data-driven approaches, leveraging interpretable rule-based intents and the expressiveness of neural networks. However, due to the timely and safety-critical nature of such models’ application, attention should be given to the resilience of the models against adversarial attacks or other uncertainties via best practices and model development and testing policies, KPIs and so on, specifically tailored to AV traffic operations [123].

Beyond their use in modeling traffic dynamics, simulation models are a fundamental tool to study and expand the ODDs of automated and connected vehicles. Advanced simulators allow researchers and operators to test the performance of vehicles beyond currently validated conditions by replicating, in a controlled manner, complex and rarely occurring traffic scenarios such as mixed traffic scenarios or sensor failures. Such a process supports the systematic identification of ODD boundaries and provides insight into how these can be safely expanded through design or policy interventions. Secondly, simulation enables the prediction of risks and the investigation of safety-critical events without putting real road users in danger, while also providing synthetic data for training and validating perception and decision-making models under different conditions [124,125].

Traffic management at complex geometries with high accident risk usually involves a local management approach for informing AVs and navigating them in a manner to avoid takeover requests (TORs), which in turn can trigger transitions of control (ToCs), or even minimum-risk maneuvers (MRMs) [126]. This may be achieved by optimizing the location of infrastructure-based solutions (e.g., RSUs) and the time to broadcast a request to the AV to follow a specific path, a speed, headway, and/or lane advice, and a request for traffic separation. The above approach does not take into consideration the driver and whether the changes in the kinematic characteristics are done in an acceptable manner (in terms of safety and comfort), nor the interactions with the VRUs that may exist in the vehicle’s scene. The interaction with vulnerable road users, such as pedestrians, is currently under research, and it gains increasing attention as a major pillar of road safety [37].

Beyond that, connectivity and automation in traffic and possible vulnerabilities of such systems coming from ODD discontinuities may have far-reaching implications not only to safety but also to the performance of the road networks. This may signify that current large-scale traffic management strategies should be revisited at least to include CAVs. The literature has for a considerable length of time tested CAVs participation to traffic flow performance and management under CAVs, including traffic control [127,128], platooning [129], speed advice and vehicle trajectory control [3,130], lane allocation and transit-aware lane allocation and control [131,132,133], auction-based intersection control [134,135], and combination of the above [136,137].

The development and testing of advanced strategies may entail certain limitations, mainly since the effects we want to observe have an impact at the microscopic level and involve multimodal interactions. Currently, this degree of connectivity and information flow in transport networks is not available. Simulation is a unique tool to scale up on the localized impacts of connectivity and automation and create scenarios with the required degree of complexity to improve and expand ODD. However, the existing simulation tools cannot cope with the problem complexity at a large scale. Furthermore, the simulation tools are plagued by a scarcity of multimodality and models capable of addressing delays resulting from interactions. This necessitates the development of a unified, modular, predictive, data-fused, and policy-driven platform, capable of accurately modeling the behavior of CAVs and their interactions with other vehicles and infrastructure.

To that end, advanced traffic simulators no longer rely on fixed, rule-based mathematical equations, such as car-following or lane-changing models. Instead, they combine these physics-based rules with data-driven learning methods that can better and more realistically reflect how real drivers and vehicles behave in different conditions [138]. Methodologies such as imitation learning, reinforcement learning, and deep generative models allow simulated vehicles to acquire driving behaviors from real-world data. In imitation learning, agents learn to imitate expert drivers by mapping observed states to actions, capturing naturalistic driving styles and interactions among vehicles [139]. Reinforcement learning expands on this by allowing agents to interact with their environment, adapt to new situations, and optimize driving strategies through reward-based feedback. More recent approaches, such as Adversarial Inverse Reinforcement Learning (AIRL) and Generative Adversarial Imitation Learning (GAIL), combine these ideas to improve robustness and diversity in simulated traffic behaviors [139,140]. Additionally, deep generative models, including Variational Autoencoders (VAEs), Generative Adversarial Networks (GANs), and diffusion models, can generate realistic and rare traffic scenarios, which are crucial for testing safety-critical events that do not appear frequently in naturalistic datasets [141,142]. Therefore, advanced traffic simulators can be a valuable tool for exploring how automated and connected vehicles operate under diverse and changing traffic, environmental, and infrastructure conditions.

Ultimately, the implementation of these modeling and control strategies requires clear institutional roles, collaborative governance, and a unified safety philosophy.

### 4.4. Organizational Frameworks to Support AV Traffic

Undoubtedly, the primary challenge lies in designing predictive and extended ODDs that facilitate cooperation with the PDI in a safe manner, allowing for improved traffic flow conditions regardless of the mix of vehicle technologies on the road. In this regard, it is crucial to strike a balance between the interests of the industry, operators, and authorities regarding the design, development, and operation of CCAM on existing networks. The industry cannot unilaterally develop and operate sustainable and robust ODDs without the input and feedback of authorities and operators. Future ODDs should be designed with numerous redundancies (fail-safe design) to accommodate increased tolerance for vehicle-specific and context-specific capabilities, human errors, and to collaborate effectively with physical and digital road infrastructure to enhance proactive safety. Road administrations and operators must be informed about the existing multiple ODDs and their adaptability to the specific conditions of each road, enabling efficient management. To achieve this, seamless, safe, and secure physical and digital environments must coexist in a resilient governance framework and collaborate to facilitate the appropriate flow of data, information, and decisions.

There is a strong absence of a unified organizational framework to support the dynamic ODD of AVs in an evolving traffic management environment. For example, what happens when conditions change, and this prohibits an AV from operating safely with respect to the rest of the traffic or vulnerable road users? How does each car maker address traffic safety, and is it possible that their approach contradicts the authorities’ or operators’ perception and regulations about safety? In view of the above, there is a strong demand for clear requirements and a safe system design framework to enable actors’ cooperation for adaptable, scalable, extendable, and secure ODDs in a continuous information flow environment for safer and trustworthy ADS. So far, however, the literature has not clearly defined the Safe System Design (SSD) as a guiding concept that all ODDs should follow, leaving a research gap in how to make sure that CCAM systems operate safely in real conditions.

Traditionally, SSD has been rooted in human-centric design, focusing on factors such as driver perception, reaction time, and error tolerance [143]. The new SSD framework should accommodate elements to support AV integration. First, seamless interaction between AVs and human drivers or other road users is crucial, especially during the transitional phase of mixed traffic, requiring infrastructure that facilitates safe and efficient coexistence [144]. Second, SSD facilitates the expansion of ODDs by creating physical infrastructure that can accommodate varying levels of automation, weather conditions, and traffic patterns [145]. Third, scalability and adaptability are essential as infrastructure must evolve alongside advancements in AV technology to ensure compatibility and long-term sustainability [146]. Finally, ethical risk mitigation through advanced technologies can improve situational awareness, support real-time decision-making, and proactively reduce potential risks.

To that end, a unified governance framework must not only align institutional roles and responsibilities but also support the integration of advanced technologies that enable real-time coordination, resilience, and adaptability. Among these, Artificial Intelligence (AI) plays a critical role, not only as a perception and prediction tool but as a cross-cutting enabler that supports decision-making and actor collaboration across all layers of the mobility system.

### 4.5. The Role of AI and Its Governance

AI and ML techniques have long constituted the computational foundation of CCAM systems, enabling perception, prediction, and control through large-scale data mining, pattern recognition, and predictive analytics. Recently, however, AI has evolved from a passive analytical component to an active decision-making interface mediating between human operators, physical assets, and digital infrastructures. This evolution redefines AI as a collaborative agent that supports situational awareness, decision coordination, and system adaptability, while simultaneously increasing the complexity of ensuring transparency, interpretability, and generalizability under diverse operational conditions. Some applications include Reinforcement Learning agents for driving recommendations [120,147], Gen AI applications on VAE and GANs in traffic perception, prediction, and decision-making within ITS [121], as well as Large Language Models (LLMs) for traffic control [148], autonomous driving [149], navigation [150], data analysis, traffic control, and scenario planning [151] and drivers’ assistance [152]. The emerging paradigm of AI-assisted actor cooperation has the potential to continuously adapt to human behavioral and cognitive states while managing operational risks through predictive simulation and scenario-based reasoning, transforming operators and users from reactive controllers to proactive supervisors who can anticipate, assess, and mitigate risks using AI-driven insights and recommendations.

From an architectural perspective, AI models used in CCAM systems are increasingly designed as modular and hierarchical structures. In such architectures, perception, prediction, decision-making, and control are implemented as separate but coordinated components [125,153]. Convolutional neural networks and transformer-based models are commonly used for perception and scene understanding, while recurrent and graph-based models are applied to trajectory prediction and interaction modeling. Reinforcement learning is frequently employed to support adaptive decision-making under uncertainty. These architectures allow models to process multimodal data and improve their ability to generalize across different traffic scenarios and infrastructure configurations [124].

Within this context, AI-assisted actor cooperation refers to the coordinated use of AI systems by vehicles, infrastructure operators, traffic managers, and human supervisors through shared situational awareness and decision support. AI systems do not replace human decision-making, but instead support it by aggregating information, predicting risks, and providing recommendations [154]. This approach is particularly relevant in mixed traffic and transitional deployment phases, where coordination between automated systems and human actors is necessary to maintain safety, efficiency, and operational continuity [153].

Despite these advancements, a limited comprehension of AI system behavior under diverse environmental and operational circumstances continues to pose a fundamental impediment to the safe and dependable deployment of CCAM. Achieving certified and trustworthy AI necessitates addressing concerns regarding generalization, training, and system failure resilience. Model development and testing should encompass the quantification of inherent and epistemic uncertainty for robust decision support, the utilization of validation metrics and performance metrics facilitating cross-model benchmarking, as well as error, confidence, and sensitivity analyses to guide optimal model updates and revision strategies. Furthermore, the credibility of AI models relies on rigorous validation, assessing the extent to which a model accurately represents real-world processes, and verification, ensuring that its implementation adheres to the intended design specifications. Evidently, when developing and operating AI systems or agents, one should think beyond xAI to ensure trustworthiness [155] and address aspects of governance acting at the entire life cycle of AI, including processes for continuous tracking, monitoring, and evaluation of model performance, auditing for traceability, explainability, validation, and verification of model results, mechanisms to report, analyze, and address risks coming from AI system failures or incidents, quality assurance mechanisms, compliance with regulatory frameworks (e.g., EU AI Act), etc. This complete approach to developing and managing AI for transport will be important to establishing resilient links between mobility and autonomous driving profiles, ensuring trustworthiness and acceptability [156,157].

Building on these, AI and ML provide the computational backbone for predictive and extensible ODDs, acting as both infrastructure-aware decision support systems and adaptive agents that coordinate information, actions, and risk management across vehicles, infrastructure, and institutional actors.

## 5. Conclusions

This paper analyzed the operational, infrastructural, and technological factors that shape and expand the ODDs of CCAM systems. The review of literature underscored that the safe and scalable deployment of AVs hinges not only on the capabilities of the vehicles themselves, but also on the readiness of the physical and digital infrastructure, simulation tools, and governance frameworks. These elements are crucial for ensuring continuous adaptation and maintaining trustworthiness. While ODD definitions are undergoing a gradual standardization process, practical implementation remains fragmented. ODD boundaries are frequently overly restrictive for scenarios involving complex and mixed traffic conditions. Conversely, broader and more flexible ODDs require infrastructure and digital ecosystems capable of dynamically adapting to environmental, traffic, and communication fluctuations.

From the infrastructure perspective, factors such as lane width, sight distance, signage retroreflectivity, and pavement condition have a very significant impact on ODD limits. Targeted upgrades and predictive maintenance strategies will be able to optimize most of these parameters for AV operations. Technological enablers such as GNSS-5G positioning, digital twins, collaborative perception, and remote operation were identified as crucial tools to improve situational awareness and ODD resilience. These technologies should be integrated within a secure, decentralized data management framework that can guarantee interoperability while protecting privacy. Similarly, simulation tools can play a significant role in extending ODDs by enabling controlled testing of rare or unsafe scenarios, risk prediction, and the training of perception models. The next generation of traffic simulators should combine physics-based dynamics with data-driven learning approaches (e.g., imitation learning, reinforcement learning, and diffusion models) to achieve realistic, adaptive, and scalable representations of automated traffic behavior.

From an organizational and governance standpoint, the study underlined the absence of a comprehensive framework that could facilitate consensus formation among industry stakeholders, operators, and regulatory authorities regarding the design, development, and operation of CCAM on existing networks. To address this, the present work proposes the adaptation of the concept of SSD to the needs of CCAM systems. The alignment of the principles of a SSD with regulatory frameworks on AI trustworthiness will be essential to ensure interoperability, and ethical operation of AI-enabled CCAM systems.

To connect the findings of this review, the proposed workflow for predictive and extendable ODD development offers a structured approach to integrating the enablers discussed throughout the paper. It links scenario-based safety validation, physical and digital infrastructure adaptation, perception and simulation technologies, and governance mechanisms into a continuous cycle of data collection, evaluation, and decision-making. This workflow supports the coordinated development of scalable and resilient ODDs, helping stakeholders move from fragmented deployments to integrated CCAM systems that adapt to real-world complexities.

Building on the insights of this review, future research should focus on addressing the identified methodological and implementation gaps, which constrain the scalability and safety of predictive and extendable ODDs. First, ODD boundaries quantification should be addressed, through the development of data-driven methods that can dynamically monitor and quantify ODD boundaries using real-world sensor data, this will enable continuous updates based on environmental and traffic feedback.

Second, research should be directed at developing common infrastructure readiness metrics that integrate both physical and digital attributes of the road environment. This would provide a consistent metric of the suitability of road segments for given levels of automation and guarantee interoperability across networks.

Third, collaborative and human-in-the-loop systems need to be explored in order to guarantee seamless cooperation between vehicles, infrastructure, and operators. Such collaborative systems will enhance situational awareness, allow adaptive control, and maintain public trust during transitional phases of mixed traffic by integrating predictive simulation with real-time human oversight.

Finally, future research should focus on evolving the SSD framework to explicitly account for automated and connected vehicle interactions within mixed traffic environments. This involves the development of measurable indicators for proactive risk mitigation, and communication reliability that can guide both infrastructure design and policy decisions. Integrating SSD principles with real-time data analytics, AI-driven monitoring, and digital twins will enable continuous safety validation and adaptive management of ODDs, ensuring that CCAM systems remain resilient, and human-centered as they scale.

## Figures and Tables

**Table 1:** Research and standards on CAVs testing and related metrics.

Category	Elements	Purpose/Insight	Sources
Testing Frameworks	Three-tier testing (simulation, closed-track, open-road)	Covers a wide range of conditions and stages of development	[7,22]
Scenario Typologies	Physics-based, traffic-critical, perception-critical, vehicle-control scenarios	Defines varied scenario origins to ensure comprehensive testing	[22]
Scenario Categorization	Hierarchical tags using ODD categories	Systematic classification to cover ODD spectrum	[21]
Data-Driven Scenario Coverage	Gaussian KDE, Coupon Collector’s Problem	Statistically ensure enough scenarios are tested	[23,24]
Scenario Creation Methods	Step-by-step scene and scenario creation	Modular building of realistic simulations	[25]
Scenario Validation & Cost	Coverage-cost tradeoff, completeness via GSN	Cost-effective planning and scenario set justification	[26,27]
ODD Coverage Metrics	Tag, time, actor-over-time coverage	Evaluates and identifies gaps in scenario datasets	[16]
Safety Metrics	MSDV, TTC, RSS, ISM, MPrISM, Criticality Metrics	Measure proximity to unsafe conditions and rule compliance	[29,30,31,32,33,34]
Advanced Safety Evaluation	α-shape and ε-almost invariant sets	Guarantees safety evaluation with limited samples	[38,40]

**Table 2:** Summary of infrastructure design factors affecting ODDs.

Factor	Influence	Standards	Literature
Stopping Sight Distance & Decision Sight Distance	Shorter distances due to lower AV perception-reaction times	-	[5,51,52]
Vertical & Horizontal Curve Design	Adjustments based on sensor placement and visibility	[9]	[5,51,54]
Lane Width	Potential reduction due to AV lateral control	[9,22,76]	[55,56]
Slopes & Grades	Need for smooth gradients to avoid sensor malfunctions	[9,76]	[55]
Speed Limits	Dynamic limits based on AV sensor capabilities	[9,22]	[52,53,57]
Tunnels	Poor GPS and light require GNSS solutions & better lighting	[22,76]	[53,54]
Bridges	Structural recalculations needed for platoons	-	[58,59]
Pavement Design	Localized wear from narrow wheel wander, fatigue	-	[61,63]
Road Markings	Primary guidance for AVs, needs reflectivity & consistency	[9]	[5,55,57,69]
Traffic Signs	Standardization and clarity are critical for AV machine vision	[9]	[67,69]
Barriers, Medians, Shoulders	Essential for separation, recovery zones, emergency stops	-	[5,70]
Lighting	Supports AV vision in low light & adverse weather	-	[56,57]
Parking Configurations	Redesign potential, space saving with AV-dedicated layouts	-	[73,75]

**Table 3:** Collaborative Perception and Maneuvering for CAVs.

Source	Collaborative Aspect	Contribution	Insights
[93]	Perception	Message filtering & congestion control for CPS	Improved scalability and network efficiency
[95]	Maneuvering	Implicit, explicit & emergent collaboration taxonomy	Framework for classifying AV coordination strategies
[92]	Perception	Pedestrian detection via RSU perception sharing	Real-world validation of V2X data improving safety
[104,105]	Maneuvering	Fast response lane changes via lateral/longitudinal split	Real-world tests of interoperable AVs
[106]	Maneuvering	V2I infrastructure supports maneuver negotiation	ETSI extension for infrastructure role
[107]	Maneuvering	MILP for optimal joint maneuver plans	Centralized computation for AV compatibility
[98]	Maneuvering	Three trajectory types for maneuver negotiation	Structured message protocol using MCM
[108]	Maneuvering	4-phase V2X lane-merging protocol	Ensures communication reliability
[100]	Maneuvering	V2V communication for highway lane changes	Controller-based maneuver protocol
[97]	Maneuvering	Trajectory conflict detection & resolution	Smooth conflict-free path planning
[103]	Maneuvering	Negotiation with persuasion in mixed traffic	Adaptive AV behavior based on human traits
[109]	Maneuvering	Protocol to align AV goals in maneuvers	Conflict-free maneuvering assurance
[101]	Maneuvering	Ten safety messages for merging	Message types for negotiation simulation
[110]	Maneuvering	Template-based maneuver exclusion logic	Robust plan generation under constraints
[111]	Maneuvering	Model-based cruise control with V2X	Markov chains for dynamic behavior modeling
[112]	Maneuvering	Game-theory for unprotected left turns	Adaptive decision-making in ambiguous cases
[113]	Maneuvering	Monte Carlo Tree Search (MCTS) for tactical planning	Smoothed multi-agent AV interactions
[102]	Maneuvering	Explicit lane-changing negotiation protocol	Structured right-of-way message exchange
[99]	Maneuvering	Cost & utility-based coordination on highways	Enhanced efficiency through optimization
[96]	Maneuvering	Cost & utility-based coordination on highways	Enhanced efficiency through optimization
[114]	Maneuvering	Common knowledge sharing before negotiation	V2V-based environmental modeling
[115]	Maneuvering	Recursive negotiation without V2X	Human-like decision-making

## Data Availability

No new data were created or analyzed in this study. Data sharing is not applicable to this article.
